# Dysregulated Marginal Zone B Cell Compartment in a Mouse Model of Sjögren’s Syndrome with Ocular Inflammation

**DOI:** 10.3390/ijms19103117

**Published:** 2018-10-11

**Authors:** Niharika Singh, Ian Chin, Paul Gabriel, Emily Blaum, Sharmila Masli

**Affiliations:** Department of Ophthalmology, Boston University School of Medicine, Boston, MA 02118, USA; nihsingh@bu.edu (N.S.); icgitar@bu.edu (I.C.); paulg@bu.edu (P.G.); eblaum@bu.edu (E.B.)

**Keywords:** marginal zone B cells, Sjögren’s syndrome, lymphoma, autoantibodies, thrombospondin

## Abstract

The risk of developing lymphoma in patients with Sjögren’s syndrome (SS) is 44 times higher than in the normal population with the most common lymphomas derived from marginal zone B (MZB) cells. Current understanding of the role of MZB cells in SS is primarily based on salivary gland pathology, while their contextual association with lacrimal glands and ocular manifestations largely remains unknown. We examined this possibility using a SS mouse model (thrombospondin-1 deficient (TSP1^−/−^)) with well-characterized ocular disease. We determined the frequency, localization, and cytokine profiles of MZB cells and their association with an antibody response in TSP1^−/−^ mice treated with a TSP-derived peptide. A significantly increased frequency of MZB cells was detected in the spleens and lacrimal glands of TSP1^−/−^ mice in comparison to wild-type tissues as detected by immunostaining. An altered cytokine profile of TSP1^−/−^ MZB cells was supportive of T helper 17 (Th17)-related pathogenesis. A significantly reduced antibody response and the splenic MZB compartment against an eye-derived antigen were noted in TSP-derived peptide-treated mice. These changes correspond with the previously reported ability of the peptide to ameliorate SS-related ocular manifestations. Collectively, our results demonstrate dysregulation of MZB cells in TSP1^−/−^ mice and highlight their role in the context of SS-related chronic ocular surface disease.

## 1. Introduction

Sjögren’s syndrome (SS) is a rheumatic disease that affects both the oral and ocular mucosa, as salivary and lacrimal glands are targeted by the autoimmune response. The main histopathological feature of SS is periductal aggregates of mononuclear cells in the glands comprised of T and B lymphocytes. Larger aggregates forming germinal center-like structures are reported in salivary glands and are derived from hyperreactive B cells associated with autoantibodies commonly detected in SS patients [1]. Studies examining the plasma cells infiltrating the salivary glands of SS patients show predominance of immunoglobulin G and M positive (IgG^+^ and IgM^+^) cells as compared to IgA^+^ cells found in normal salivary glands [2]. Non-Hodgkin’s B-cell lymphoma is a most serious complication of SS with the most common being low-grade marginal zone lymphomas [3]. While the risk of developing such lymphomas is equal in primary and secondary SS patients, it is 44 times greater than that observed in the normal population. Marginal zone B (MZB) cells were reported in the lymphocyte infiltrates of salivary glands in SS patients and are implicated in local production of autoantibodies and subsequent glandular destruction [4]. However, the presence of MZB cells in lacrimal glands during SS remains to be elucidated.

Unlike circulating follicular (FO) B cells, MZB cells are an innate subset localized in distinct areas surrounding B-cell follicles in secondary lymphoid organs [5]. Phenotypically, MZB cells express reduced levels of negative regulator of B cells, cluster of differentiation 23 (CD23; CD23^low^), which is consistent with their ability to produce a rapid antibody response compared to FO B cells. Their expression of polyreactive B-cell receptor (BCR) aids efficient primary antibody response against soluble protein antigens, independently of T-cell help. As such, MZB cells are an integral part of innate immunity. Additionally, MZB cells are also reported to be efficient antigen-presenting cells (APCs) that effectively prime CD4^+^ T cells [6]. Enrichment of autoreactive specificities in the MZB compartment and their participation in innate and adaptive immune responses implicated MZB cells in the development of autoimmune pathology [7].

In humans, in addition to secondary lymphoid organs, MZB cells are also found in the ectopic mucosa-associated lymphoid tissue (MALT) located at sites that include the salivary gland, lacrimal duct, and conjunctiva [8,9]. Detection of autoreactive MZB cells in salivary glands of SS patients is proposed as closely related to lymphoma development [10]. Considering that normal checkpoint mechanisms in secondary lymphoid organs eliminate autoreactive MZB cells, it was suggested that ectopic lymphoid tissues represent a reservoir of self-reactive MZB cells that escape normal elimination [1]. In mouse models of autoimmune diseases, an increased frequency of splenic MZB was reported [11,12,13], and, particularly in SS models, the transgenic elimination of MZB cells was reported to eliminate salivary and lacrimal gland pathology [14,15]. Therefore, emerging evidence lends support for an important role of MZB cells in SS pathogenesis and suggests MZB cells as a potential therapeutic target in SS. However, the distribution of MZB cells in the context of ocular phenotype in SS and their contribution to pathology at a functional level are not known.

In this study, we used a thrombospondin-1 deficient (TSP1^−/−^) mouse model of SS that develops ocular manifestations similar to those seen in SS patients [16,17]. Systemically, these mice develop SS-specific autoantibodies with a peripheral imbalance in regulatory and inflammatory effectors. Moreover, we reported that a topical application of a TSP-derived peptide ameliorates SS-associated ocular inflammation in TSP1^−/−^ mice [18]. To better understand the function of MZB cells in the context of SS pathogenesis and ocular manifestations, we examined MZB distribution in TSP1^−/−^ mice and their function. Our results provide the evidence that the MZB compartment is enlarged and dysregulated in TSP1^−/−^ mice, the increased MZBs contribute to antibody response against eye-derived antigens, and they are regulated by the TSP-derived peptide known to ameliorate SS-related ocular symptoms.

## 2. Results

### 2.1. Splenic Marginal Zone Compartment Is Altered in TSP1^−/−^ Mice

**Figure 1:**
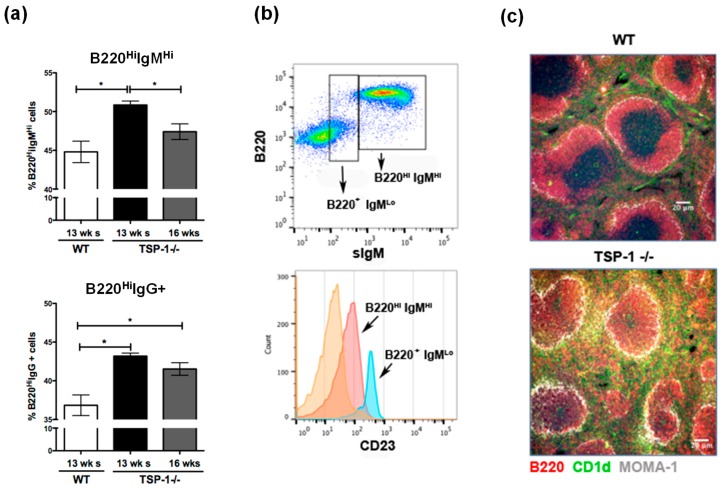
Frequency of marginal zone B (MZB) cells is increased in thrombospondin-1 deficient (TSP1^−/−^) spleens. (**a**) Frequency of B220^Hi^ cells in the spleens of wild-type (WT) and TSP1^−/−^ mice of indicated ages as detected that had high levels of immunoglobulin M (IgM^Hi^) or IgG^+^ by flow cytometric analysis. (**b**) Levels of cluster of differentiation 23 (CD23) expression in splenic B220^+^ subsets. (**c**) Intrasplenic structural differences between WT and TSP1^−/−^ mice in the distribution of MZB cells (yellow) identified by immunostaining for B220 (red) and CD1d (green). Metallophilic macrophages are identified by anti-metallophilic macrophages antibody (MOMA-1; grey white) staining around follicles (100×; scale bar, 20 µm; * *p* < 0.05).

Sjögren’s syndrome-specific autoantibodies, anti-SSA and anti-SSB, are detectable in 12–16-week-old TSP1^−/−^ mice [16]. To determine if the B-cell phenotype in their spleens correlates with the autoimmune pathology, we examined surface IgM and IgG expression on B cells (B220^+^) in the spleens harvested from 13-week-old wild-type (WT) or TSP1^−/−^ mice. Flow cytometric analysis of immunostained cells was performed to determine the percentage of B220^+^ cells stained positively for the surface IgM or IgG. As shown in Figure 1a, significantly increased numbers of both IgM- and IgG-expressing cells were detected in TSP1^−/−^ mice compared to WT mice. Moreover, at 16 weeks of age, a significant decline in IgM-expressing cells was noted in TSP1^−/−^ mice compared to those detected at 13 weeks of age. However, numbers of IgG-expressing cells remained elevated in older TSP1^−/−^ mice suggesting a potential class switch with progressing age. Considering that MZB cells express high levels of surface IgM [5], we next determined if B220^Hi^IgM^Hi^ cells expressed other markers associated with MZB cells like CD23 [19]. As shown in Figure 1b, B220^Hi^IgM^Hi^ cells expressed lower levels of CD23 as seen in MZB cells. The proportion of such MZB cells is significantly increased in other autoimmune disease models [13,20]. To confirm changes noted in flow cytometric analysis, we examined frozen sections of spleens harvested from WT or TSP1^−/−^ mice immunostained for B220^+^CD1d^+^ MZB cells. As shown in Figure 1c, the marginal zone around B-cell follicles was clearly marked by anti-metallophilic macrophages antibody (MOMA-1^+^; (grey white) in both WT and TSP1^−/−^ spleens. Consistent with an increased proportion of IgM^hi^ cells detected by flow cytometry, an enlarged B220^+^CD1d^+^ (yellow) MZB cell compartment was detected around the marginal zone in the TSP1^−/−^ spleen as compared to the WT spleen. Additionally, in TSP1−/−, but not WT spleens, MZB cells were also detected in intrafollicular areas as reported in other autoimmune disease [20]. Thus, overall, significant alterations of the splenic marginal zone are seen in TSP1^−/−^ spleens, due to an enlarged MZB compartment and an encroachment of MZB cells into the follicular area. These results suggest that MZB cells likely contribute to the SS pathology in TSP1^−/−^ mice.

### 2.2. Frequency of MZB Cells Is Increased in the Lacrimal Glands of TSP1^−/−^ Mice

**Figure 2:**
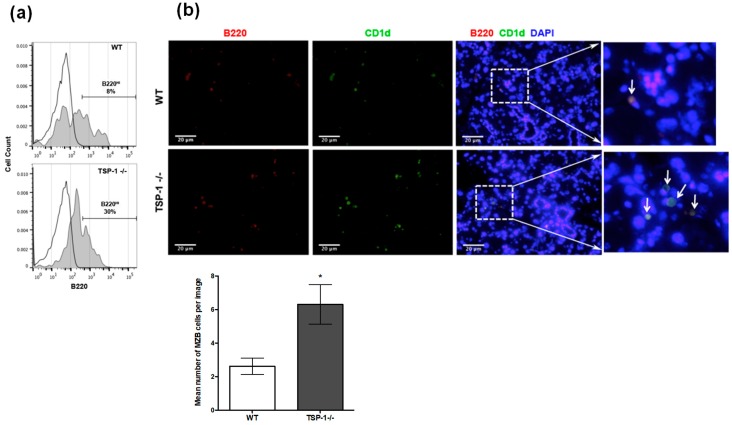
Frequency of MZB cells is increased in the lacrimal glands of TSP1^−/−^ mice. (**a**) Mononuclear cells from lacrimal glands of 12-week-old WT or TSP1^−/−^ mice were stained for CD45 and B220 and analyzed by flow cytometry. Histograms (normalized plots to unit area) represent B220 staining of CD45^+^ cells. Frequencies of B220^Hi^ cells that include MZB cells are indicated. (**b**) Representative images of immunostained lacrimal gland sections from WT and TSP1^−/−^ mice (200×; scale bar, 20 µm). Frozen tissue sections were stained for B220 (red) and CD1d (green). Nuclei were stained with 4′,6-diamidino-2-phenylindole (DAPI; blue). Far right panels show magnification of areas marked by boxes. Arrows indicate B220^+^CD1d^+^ MZB cells. The bar graph shows the quantitative representation of the immunostaining with mean number of MZB cells (enumerated from six images from different areas per group) +/− SEM; * *p* < 0.05.

**Figure 3:**
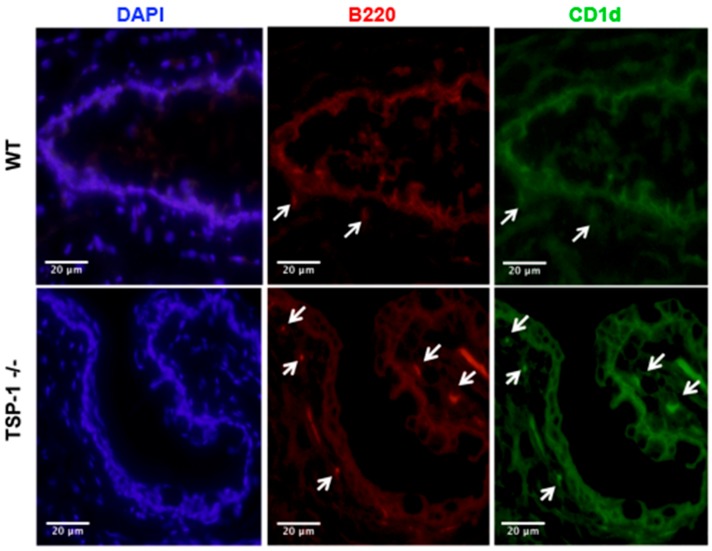
Detection of MZB cells in the conjunctiva (ocular mucosal tissue). Representative images of immunostained sections of conjunctiva from WT and TSP1^−/−^ mice (200×; scale bar, 20 µm) are shown. Frozen tissue sections were stained for B220 (red) and CD1d (green). Nuclei were stained with DAPI (blue). Cells positive for both B220 and CD1d represent MZB cells and are marked with white arrows.

It is established that the lacrimal glands are targeted by the autoimmune response in SS, but it is not known if more MZB cells infiltrate the lacrimal glands of murine models of SS. We assessed the frequency of MZB cells in the lacrimal glands of TSP1^−/−^ mice compared to WT mice by harvesting mononuclear cells from collagen-digested lacrimal glands and analyzing B220 expression of CD45^+^ cells by flow cytometry. As shown in Figure 2a, increased numbers of B220^Hi^ cells that include MZB cells are detected in TSP1^−/−^ lacrimal glands as compared to the WT tissue. We next immunostained frozen sections of lacrimal glands to detect B220^+^CD1d^+^ MZBs as detected in the spleens. Consistent with the flow cytometric data, significantly more MZB cells were detected in the lacrimal glands of TSP1^−/−^ mice compared to the WT glands (Figure 2b). In fact, similar B220^+^CD1d^+^ MZB cells were also detected in the conjunctiva tissue (Figure 3) with relatively increased numbers in TSP1^−/−^ mice compared to WT mice. These results indicate that more MZB cells are found in the lacrimal glands and conjunctiva, tissues relevant to the ocular phenotype characterized in TSP1^−/−^ mice, suggesting that MZB cell localization in these tissues is associated with the pathogenesis of the disease.

### 2.3. Cytokine Secretion by MZB Cells Is Altered in TSP1^−/−^ Mice

**Figure 4:**
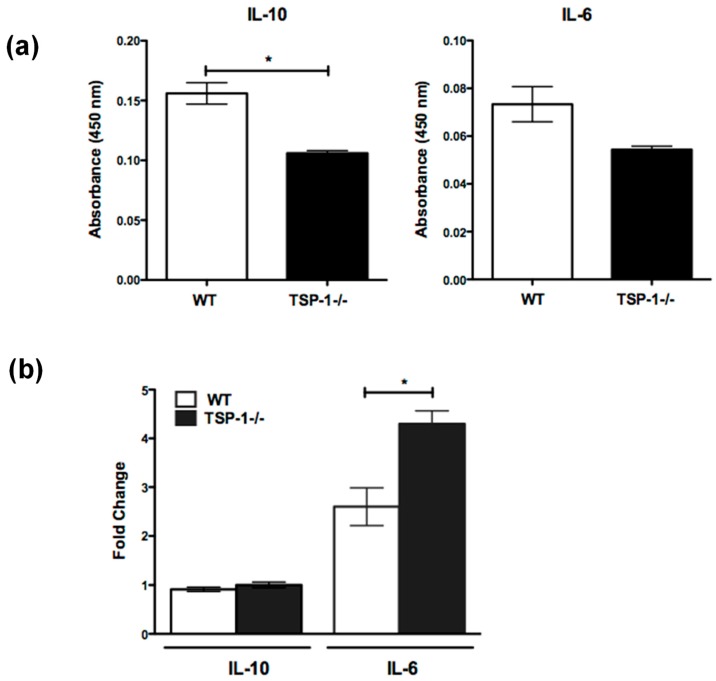
Cytokine secretion by MZB cells from TSP1^−/−^ mice is altered. Sorted splenic MZB (CD43^−^CD23^−^CD21^+^) cells were cultured for three days in the absence (**a**) or presence (**b**) of apoptotic cells and cytosine–phosphate–guanine (CpG-B). Culture supernatants were evaluated for the levels of interleukins 10 and 6 (IL-10 and IL-6) using ELISA. Results show mean and standard error of the mean (SEM) values (*n* = 3 per strain from two independent sorts). In the case of stimulated MZB, results are presented as cytokine levels relative to unstimulated cells; * *p* < 0.05.

In addition to being associated with autoantibody secretion, MZB cells are reported to contribute to immunologic tolerance by secreting immunosuppressive cytokine interleukin 10 (IL-10) [21]. Moreover, MZB-derived IL-6 is reported to play an essential role in the propagation of T helper 17 (Th17) response [22]. Considering the previously reported peripheral imbalance between regulatory T cells (Treg) and inflammatory effectors, including the increased frequency of Th17 in TSP1^−/−^ mice, we assessed the ability of their MZB cells to secrete IL-10 and IL-6. We first determined spontaneous secretion of these cytokines by splenic MZB cells (CD43^−^CD21^+^CD23^−^) derived from WT or TSP1^−/−^ mice. As shown in Figure 4a, in culture supernatants derived from TSP1^−/−^, as compared to WT, MZB cells contained significantly reduced levels of IL-10, but there was no difference in IL-6 levels. Marginal zone B cells are known to respond strongly via their B-cell receptors (BCRs), as well as Toll-like receptors (TLRs) [19]. In particular, DNA-containing chromatin complexes on the surfaces of apoptotic cells can engage TLR-9 receptors on MZB cells to activate them [23]. We reported significantly increased apoptosis in the lacrimal glands of TSP1^−/−^ mice [16]. Based on the increased frequency of MZB cells in the lacrimal glands of these mice, we assessed their cytokine secretion in response to stimulation via TLR-9 ligands, cytosine–phosphate–guanine (CpG) sites, and apoptotic cells. Our results demonstrate that TSP1^−/−^ MZB cells respond to such stimuli by secreting significantly increased levels of IL-6 as compared to similarly stimulated WT cells (Figure 4b). Such a difference was not noted in IL-10 secretion. Together, these results suggest that MZB cells in TSP1^−/−^ mice express an altered cytokine IL-10 and IL-6 profile that is consistent with their increased Th17 frequency and autoimmune pathology.

### 2.4. Thrombospondin-Derived Peptide Reduces Antibody Response and Splenic MZB Compartment Size in Immunized TSP1^−/−^ Mice

**Figure 5:**
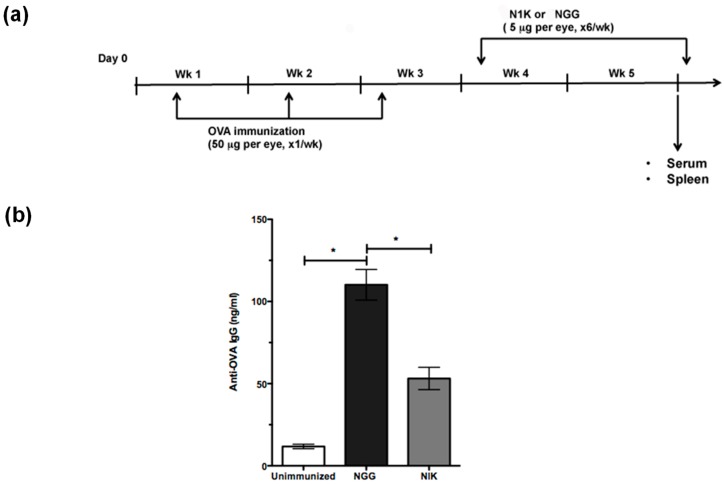
Topically administered TSP-derived peptides attenuate ovalbumin-specific antibody response in immunized TSP1^−/−^ mice. (**a**) Timeline of ovalbumin (OVA) immunization and subsequent treatment of TSP1^−/−^ mice with TSP1-derived peptide (N1K) or control peptide (NGG). (**b**) Serum anti-OVA IgG levels as determined by ELISA; Mean Ab level +/− SEM. Data are representative of two independent experiments; * *p* < 0.05.

**Figure 6:**
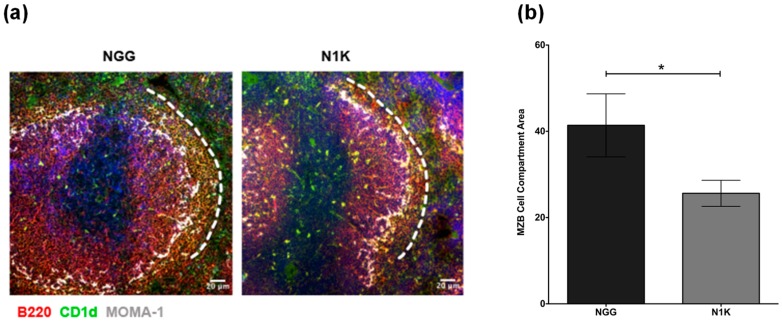
Topically administered TSP1-derived peptides decrease the area of splenic MZB cells in immunized TSP1^−/−^ mice. (**a**) Representative images of frozen sections of spleen from N1K- and NGG-treated TSP1^−/−^ mice (100×; scale bar, 20 µm). Sections were immunostained for MZB cells (B220^+^CD1d^+^)-B220 (red), CD1d (green), MOMA-1 (grey white), and nuclear stain DAPI (blue). The MOMA-1^+^ (grey white) macrophages around follicles and the white dotted line mark the inner and outer boundaries of the MZB cell compartment, respectively. (**b**) Quantitative assessment of area covered by MZB cells determined using the ImageJ software as described in Section 4.4. Five to six images representing independent areas and animals per experimental group were analyzed; Data represents Mean +/− SEM; * *p* < 0.05.

Autoimmune pathology typically represents a T-dependent antibody response that involves germinal center (GC) formation with early extrafollicular T-independent activation of B cells [24]. MZB cells located immediately outside follicles are known to efficiently present soluble antigens to CD4 T cells [6]. Therefore, it is possible that MZB cells present eye-derived antigens to generate an immune response. To test this possibility, we used model T-dependent soluble protein antigen ovalbumin (OVA) as a surrogate to immunize TSP1^−/−^ mice, as specific eye-derived antigens remain to be identified. We treated these immunized mice with a TSP-derived peptide (N1K) that was reported to ameliorate ocular manifestations in TSP1^−/−^ mice [18]. Mice in the control group were immunized and treated with an inactive control peptide (NGG). The immunization and topical treatment schedule is outlined in Figure 5a. At the end of the study period, serum samples from the mice were analyzed for levels of OVA-specific IgG antibodies in an ELISA. A corresponding change in splenic MZB compartment was examined by immunostaining. As shown in Figure 5b, the anti-OVA IgG response generated by immunization via topically applied OVA was significantly reduced in mice treated with N1K compared to the control group treated with NGG. This reduced antibody in N1K-treated mice correlated with the significantly reduced area occupied by MZB cells in their spleens as compared to those from the control group (Figure 6). Collectively, these results support a strong potential of MZB cells in generating an immune response against eye-derived antigens and suggest their potential contribution to SS-associated ocular manifestations in TSP1^−/−^ mice.

## 3. Discussion

Increased numbers of IgM^+^ memory B cells detected in the periphery and salivary glands of SS patients were identified as MZB cells [4,25], which are implicated in local antibody production and subsequent destruction of glandular epithelial cells. Similarly, MZB cells in salivary glands of several SS mouse models correspond with salivary hypofunction [26]. However, only limited studies addressed the presence of B cells in lacrimal gland pathology of SS [27,28]. In this study, we report the spontaneously increased frequency of MZB cells in the spleens and lacrimal glands of TSP1^−/−^ mice that were extensively characterized for their SS-related ocular disease. Our results demonstrate that the altered cytokine profile of MZB cells in these mice correlates with their autoimmune pathology and their potential to generate antibodies against eye-derived antigens. Together, our results suggest a significant association of MZB cells with chronic SS-related ocular pathology. Moreover, our results demonstrate that a therapeutic approach that successfully ameliorates ocular inflammation and Th17 response in TSP1^−/−^ mice also reduces MZB frequency. Thus, MZB cells likely represent a common therapeutic target that addresses SS pathology in both exocrine glands.

In this study, we report the significantly increased frequency of B cells expressing high levels of IgM in TSP1^−/−^ mice. This observation is analogous to that reported in SS patients. However, in older TSP1^−/−^ mice, the frequency of IgM^Hi^ B cells declined, while the frequency of IgG^+^ B cells remained elevated, presumably due to class switching among activated B cells. The IgM^Hi^ cells in TSP1^−/−^ mice appeared to be MZB cells based on their lower expression of CD23. The enlargement of the MZB compartment was confirmed with the detection of increased frequency of B220^+^CD1d^+^ cells at a unique anatomical location in the splenic marginal zone area, clearly marked by marginal MOMA-1^+^ macrophages at the follicular boundary. Such an MZB expansion is associated with autoimmunity and is consistent with findings reported in other mouse models of SS [26]. It is possible that increased MZB frequency results from augmented antigenic stimulation of either self or non-self origin. These cells are also reported to migrate to extrasplenic locations, such as the salivary gland, as a disease-related target organ in SS patients, as well as mouse models [4,29]. Consistent with these reports, we detected MZB cells in the lacrimal gland, as well as conjunctiva, of TSP1^−/−^ mice, suggesting their relevance in SS-related ocular pathology.

The expansion of MZB cells in the spleen and their presence in the lacrimal gland and conjunctiva of 12-week-old TSP1^−/−^ mice suggests a possibility that autoantigens derived from ocular surface-related tissues contribute to the activation of MZB cells and subsequent antibody responses. In addition to responding to T-independent antigens, MZB cells are also potent antigen presenters to CD4^+^ T cells and, as such, can induce antigen specific immune responses against T-dependent antigens [6]. Consistently, migration of autoreactive MZB cells to intrafollicular regions and their interactions with CD4^+^ T cells are reported to contribute to autoimmune process [20]. Similarly, our results demonstrate altered splenic architecture in TSP1^−/−^ mice where, unlike in WT spleens, MZB cells are not restricted to the marginal zone. Their intrafollicular location is consistent with the autoimmune pathology in TSP1^−/−^ mice and supports their potential involvement in T-dependent responses. Thus, the expansion of the MZB compartment in the TSP1^−/−^ mouse model is consistent with the increased numbers of MZB cells reported in other mouse models of SS (non-obese diabetic (NOD), C57BL/6.NOD-Aec1Aec2, and IL-14Tg mice) in the context of salivary gland pathology [26].

Chronic stimulation by exogenous or autologous antigens, as well as over expression of B-cell survival/activation factors (B-cell activating factor (BAFF) and A proliferation-inducing ligand (APRIL)), are believed to drive B-cell hyperactivity and their subsequent transformation into lymphoma [30]. We previously reported an increased apoptosis of the lacrimal epithelial cells in TSP1^−/−^ mice [16]. It is conceivable that such apoptotic cells serve as a source of autologous antigens that provides chronic stimulation to MZB cells in these mice. In our experiments, ocular instillation of an exogenous T-dependent antigen, OVA, in TSP1^−/−^ mice led to increased serum levels of anti-OVA IgG consistent with their expanded MZB cell compartment. This OVA-specific antibody response was significantly reduced when immunized mice were treated with a TSP-derived peptide that was previously reported to attenuate ocular manifestations and Th17 responses in TSP1^−/−^ mice [18]. The reduction in circulating anti-OVA antibody levels in our experiments were also accompanied by the reduced size of the splenic MZB compartment, suggesting a potential involvement of MZB cells in generating an anti-OVA response, and therefore, eye-derived antigens. Studies reported that Th17 cells support autoreactive germinal center (GC) formation and antibody production with immunoglobulin class-switch recombination [31,32]. Thus, the presence of Th17 cells in TSP1^−/−^ mice is closely linked to the expanded MZB compartment. Therefore, we believe the effect of TSP-derived peptides on the reduced MZB compartment is likely achieved indirectly via the inhibition of Th17 effectors as reported previously [18].

Under normal conditions, the ability of MZB cells to secrete IL-10 is considered critical for the maintenance of tolerance [21,23] and for restraining autoantibody production, as the loss of IL-10 in mice resulted in MZB cell expansion and increased production of IgM autoantibodies [33]. Consistently, reduced IL-10 secretion by TSP1^−/−^ MZB cells in our study correlates with their expansion. As innate sensors, MZB cells are equipped with Toll-like receptors, TLR4 and TLR9, that compliment their stimulation via BCRs, inducing their proliferation and maturation with the release of cytokines [5]. In response to TLR9 stimulation, we noted significantly increased IL-6 secretion by TSP1^−/−^ MZB cells. This observation is consistent with the previously reported presence of Th17 cells in TSP1^−/−^ lacrimal glands [16], as MZB-derived IL-6 is known to contribute to enhanced pathogenic Th17 responses associated with autoimmune disease [22]. The appearance of Th17 cells in lacrimal glands of TSP1^−/−^ mice follows an earlier increase in apoptosis of glandular cells [16]. It is possible that these apoptotic cells stimulate local MZB cells via TLR9 ligation and support local differentiation of Th17 cells by secreting IL-6. We ruled out a direct effect of TSP-derived peptides on the cytokine profile of MZB cells, as stimulating them in the presence of the peptide did not alter their cytokine profile (data not shown). Furthermore, in our experiments, a lack of IL-10 secretion by TLR9-stimulated MZB may be due to a distinct signal requirement via TLR4 for maximal IL-10 secretion as reported by others [22].

Overall, our study demonstrates a dysregulated MZB compartment in a SS mouse model with ocular symptoms that involves MZB expansion accompanied by extrasplenic localization to the lacrimal gland and conjunctiva. Our results suggest that MZB-derived increased IL-6 likely supports pathogenic Th17 effectors, which in turn promote MZB expansion and antibody response. Such a response may include antibodies directed against antigens originating from ocular tissues. Furthermore, our results demonstrate that a therapeutic approach that successfully attenuates ocular symptoms and inhibits Th17 response indirectly targets MZB cells. Thus, our results also highlight a promising potential of anti-IL-17 therapeutics in the management of MZB lymphomas in SS patients.

## 4. Materials and Methods

### 4.1. Mice

C57BL/6 male mice (10 to 20 weeks old), were purchased from Charles River Laboratories (Wilmington, MA, USA). A breeding pair of B6.129S2-Thbs1<tmlHyn>/J (TSP1^−/−^) mice was purchased from Jackson Laboratories (Bar Harbor, ME, USA) and bred in-house at a pathogen-free facility at Boston University School of Medicine Laboratory Animal Science Center.

The Institutional Animal Care and Use Committee (IACUC) at Boston University School of Medicine, Boston, approved the animal studies described in this manuscript (Protocol number AN-15400, 30 September 2016) in accordance with the National Institutes of Health (NIH) guide for the care and use of laboratory animals. All animal experiments were conducted in accordance with the Association for Research in Vision and Ophthalmology (ARVO) statement for the Use of Animals in Ophthalmic and Vision Research.

### 4.2. Antibodies and Reagents

Anti-CD45R/B220 and fluorescein isothiocyanate (FITC)-conjugated anti-CD1d were obtained from BD Pharmingen (San Jose, CA, USA). Alexa 568 anti-rat IgG was obtained from Thermofisher (Waltham, MA, USA), while Alexa 647 conjugated anti-MOMA-1 was purchased from Bio-rad (Hercules, CA, USA). Biotin-conjugated anti-CD43, biotin-conjugated anti-CD23, and FITC-conjugated anti-CD21/35 were provided by PharMingen (San Diego, CA, USA). Avidin-phycoerythrin (PE)-Cy7 was obtained from eBioscience (San Diego, CA, USA) and PE/Cy7-conjugated anti-CD4 was provided by Biolegend (San Diego, CA, USA). TSP1-derived peptide 4N1K (KRFYVVMWKK) and 4NGG (KRFYGGMWKK) were synthesized by Bio Basic (Markham, ON, Canada), and ovalbumin (Sigma, St. Louis, MO, USA) was reconstituted in sterile phosphate-buffered saline (PBS). TLR9 ligand CpG-B oligodeoxynucleotide (ODN) was purchased from Invivogen (San Diego, CA, USA). Enzyme-linked immunosorbent assay (ELISA) kits were used to quantify IL-6 and IL-10 (eBioscience, San Diego, CA, USA) and anti-ovalbumin IgG (Chondrex, Redmond, WA, USA). Culture medium Rosewell Park Memorial institute (RPMI-1640), 10 mM 4-(2-hydroxyethyl)-1-piperazineethanesulfonic acid (HEPES), 0.1 mM non-essential amino acid (NEAA), 1 mM sodium pyruvate, 100 U/mL penicillin, 100 mg/mL streptomycin, and 200 mM l-glutamine were purchased from Lonza (Basel, Switzerland), and were prepared with fetal bovine serum (FBS; Atlanta Biologicals, Lawrenceville, GA, USA) and dexamethasone (Sigma-Aldrich, St. Louis, MO, USA).

### 4.3. Immunization and Peptide Treatment

TSP1-deficient female mice at the age of 10 weeks were immunized via topically applied ovalbumin (100 µg/mouse), bilaterally (50 µg/eye), once a week for three weeks. Immunized mice (*n* = 6 per group) were treated topically with control (NGG) or TSP-derived peptide N1K (10 µg/mouse), bilaterally (5 µg/eye), six days per week for two weeks. Serum and spleen samples were harvested at the end of the study.

### 4.4. Immunohistochemistry

Harvested tissues from mice were immediately embedded in optimal cutting temperature medium (Sakura Finetek USA, Inc. Torrance, CA, USA) in molds (Thermo Fisher Scientific, Waltham, MA, USA) and flash-frozen in a liquid nitrogen bath and stored at −20 °C. Sectioning was performed on a cryostat (Microm HM 525, Microm International GmbH, Walldorf, Germany) to generate 8-µm sections. Sections were air-dried for 15 min at room temperature, fixed in acetone for 10 min at −20 °C, and then allowed to air-dry for 10 min. Upon rehydration in Tris-buffered saline (TBS) with 0.05% Tween-20 (TBS-T) for 5 min, sections were blocked for 1 h at room temperature with TBS-T 1%, bovine serum albumin (BSA), and 4% goat serum. Further staining with primary antibodies against CD45/B220, MOMA-1, and CD1d were performed overnight at 4 °C. Sections were rinsed with TBS-T and stained with fluorochrome-conjugated secondary antibody and mounted with 4′,6-diamidino-2-phenylindole (DAPI) containing mounting medium. Negative controls were stained similarly with omission of all primary antibodies.

Stained sections were evaluated using the 10× objective on the Nikon Deconvolution Wide-Field Epifluorescence microscope using the same exposure time, gain, and intensity. The ImageJ software (NIH, Bethesda, MD, USA) was used to determine the MZB compartment area by calculating the ratio of the mean grey value for the color channel, corresponding to the MZB cells, to that obtained from the blue channel, corresponding to the DAPI-stained nuclei.

### 4.5. Flow Cytometry

Single-cell suspensions were prepared from spleens or collagen-digested lacrimal glands as described previously [16]. Cells were stained for surface markers B220, IgM, CD43, CD23, and CD21 after blocking Fc receptors with anti-Fc antibody. Respective isotype-matched antibodies were used in negative controls. Stained cells were analyzed by flow cytometry (BD LSR II; BD Biosciences at Boston University Core Services). Flow cytometric analysis was performed using the FlowJo software (Treestar, Inc, Ashland, OR, USA). Marginal zone B cells were sorted from purified CD43^−^ B cells as CD21^+^CD23^−^ using a BD FACSARIA II SORP flow cytometer (Boston University Core services).

### 4.6. Marginal Zone B Cell Assay

Apoptotic thymocytes were prepared as described by Sang et al. [34]. Briefly, thymocytes were suspended in 1 µM dexamethasone for four hours at 37 °C. The cells were then washed and resuspended in complete RPMI-1640. Marginal zone B cells were co-cultured with thymocytes at a 1:7 ratio and CpG-B (1 µg/mL). In some wells, MZB cells were stimulated with CpG-B (1 µg/mL) in the presence of 4N1K/4NGG (10 µM). Culture plates were incubated for three days at 37 °C. After incubation, the culture supernatants were collected and stored at −20 °C until ELISA analysis. ELISA kits were used to quantify IL-6 and IL-10 from the culture supernatants according to the manufacturer’s instructions.

### 4.7. Statistical Analysis

Data analysis was performed with the GraphPad Prism 6.0 software, La Jolla, CA, USA. The unpaired Student’s *t*-test was used to determine significant differences between mean values of experimental and control groups. Error bars enclosing mean values in figures represent ± standard error of the mean (SEM). A *p*-value < 0.05 was considered statistically significant.

## Abbreviations

SS	Sjögren’s Syndrome
MZB	Marginal zone B cells
TSP	Thrombospondin
Th17	T helper 17
